# Effects of Bofu-Tsusho-San on Diabetes and Hyperlipidemia Associated with AMP-Activated Protein Kinase and Glucose Transporter 4 in High-Fat-Fed Mice

**DOI:** 10.3390/ijms151120022

**Published:** 2014-11-04

**Authors:** Cheng-Hsiu Lin, Yueh-Hsiung Kuo, Chun-Ching Shih

**Affiliations:** 1 Department of Internal Medicine, Feng-Yuan Hospital, Ministry of Health and Welfare, Fengyuan District, Taichung City 42055, Taiwan; E-Mail: keny71@pchome.com.tw; 2 Department of Chinese Pharmaceutical Sciences and Chinese Medicine Resources, China Medical University, Taichung City 40402, Taiwan; E-Mail: kuoyh@mail.cmu.edu.tw; 3 Department of Biotechnology, Asia University, Taichung City 41354, Taiwan; 4 Graduate Institute of Pharmaceutical Science and Technology, College of Health Science, Central Taiwan University of Science and Technology, No. 666, Buzih Road, Beitun District, Taichung City 40601, Taiwan

**Keywords:** Bofu-tsusho-san, AMP-activated protein kinase phosphorylation, glucose transporter 4

## Abstract

This study was undertaken to examine the effect and mechanism of Bofu-tsusho-san formula (BO) on hyperglycemia and hyperlipidemia and in mice fed with a high-fat (HF) diet. The C57BL/6J mice were received control/HF diet for 12 weeks, and oral administration of BO (at three doses) or rosiglitazone (Rosi) or vehicle for the last 4 weeks. Blood, skeletal muscle and tissues were examined by means of measuring glycaemia and dyslipidaemia-associated events. BO treatment effectively prevented HF diet-induced increases in the levels of triglyceride (TG), free fatty acid (FFA) and leptin (*p* < 0.01, *p* < 0.01, *p* < 0.01, respectively). BO treatment exhibited reduced both visceral fat mass and hepatic triacylglycerol content; moreover, BO treatment displayed significantly decreased both the average area of the cut of adipocytes and ballooning of hepatocytes. BO treatment exerted increased the protein contents of glucose transporter 4 (GLUT4) in skeletal muscle, and caused lowered blood glucose levels. BO treatment displayed increased levels of phosphorylated AMP-activated protein kinase (AMPK) in both skeletal muscle and liver tissue. Furthermore, BO reduced the hepatic expression of glucose-6-phosphatase (G6Pase) and phosphenolpyruvate carboxykinase (PEPCK) and glucose production. Therefore, it is possible that the activation of AMPK by BO leads to diminished gluconeogenesis in liver tissue. BO increased hepatic expressions of peroxisome proliferator-activated receptor α (PPARα), whereas down-regulating decreasing expressions of fatty acid synthesis, including sterol regulatory element binding protein 1c (SREBP1c) and fatty acid synthase (FAS), resulting in a decrease in circulating triglycerides. This study originally provides the evidence that amelioration of dyslipidemic and diabetic state by BO in HF-fed mice occurred by regulation of GLUT4, SREBP1c, FAS, PPARα, adiponectin and AMPK phosphorylation.

## 1. Introduction

Diabetes mellitus type 2 is a metabolic disorder that is characterized by hyperglycemia in the context of insulin resistance and lack of insulin. Type 2 diabetes makes up about 90% of cases of diabetes. It is predicted that the world’s population of type 2 diabetes would be reached 6.1% by 2025 [1]. Therefore, the approaches to treatment of type 2 diabetes mellitus become to be an important issue. Central to this metabolic condition is altered glucose and lipid metabolism resulting from peripheral tissues. Insulin resistance is characterized by a decrease in insulin-stimulate glucose uptake in skeletal muscle via glucose transporter 4 (GLUT4) and by impaired suppression of glucose production in liver tissue [2]. Therefore, finding strategies to improve peripheral insulin sensitivity is important as a means to control diabetes, hyperlipidemia and complications.

Bofu-tsusho-san (BO; [Fang-feng-tong-sheng-san]), a traditional Chinese herbal remedy, has been shown to exert various beneficial pharmacological effects on atherosclerosis [3], obesity [4] and hyperglycemia [5] in Japan, Korea and China. In particular, BO has already been used as an anti-obesity drug in some local clinics of Taiwan. BO is composed of eighteen medicinal plants (Table 1). Its constituents including *Ephedra sinica*, *Platycodon grandiflorum*, *Forsythia suspensa* and *Paeonia lactiflora* have been shown to exert anti-obesity, anti-diabetes and lipid-lowering activity [6,7,8,9]. Oleanolic acid from *Forsythia suspensa* is demonstrated to decrease the blood glucose in streptozotocin-induced diabetes mice [9]. Phytosterols from *Schizonepetae Spica* exert anti-diabetic activity in streptozotocin-induced mice [10]. Platycodon (contained in Platycodi Radix) has been used as a folk remedy for hyperlipidemia and diabetes in Korea [7,11]. Paeoniae Radix is reported to exhibit the antidiabetic properties by stimulating glucose uptake while suppressing hepatic gluconeogenesis via regulating PEPCK transcription in streptozotocin-induced diabetic rats and *db/db* mice [8]. However, the effect and mechanism of BO on diabetes and hyperlipidemia remains obscure.

**Table 1:** Components of Bofu-tsusho-san (BO). 9 g extract of the above raw materials ^a^. See Materials and Methods for details.

(1) Scutellariae Radix (Labiatae)	2.0
(2) Glycyrrhizae Radix (Leguminosae)	4.0
(3) Platycodi Radix (Campanulaceae)	2.0
(4) Atractyloids Lanceae Rhizoma (Compositae)	1.0
(5) Rhei Rhizoma (Polygonaceae)	1.0
(6) Schizonepetae Spica (Labiatae)	1.0
(7) Gardeniae Fructus (Rubiaceae)	1.0
(8) Paeonia Radix (Paeoniaceae)	1.0
(9) Cnidii Rhizoma (Umbelliferae)	1.0
(10) Angelicae Radix (Umbelliferae)	1.0
(11) Menthae Folium (Labiatae)	1.0
(12) Saposhnikoviae Radix (Umbelliferae)	1.0
(13) Ephedrae Herba (Ephedraceae)	1.0
(14) Forsythiae Fructus (Oleaceae)	1.0
(15) Zingiberis Rhizoma (Zingiberaceae)	2.0
(16) Gypsum Fibrosum	2.0
(17) Natrium Sulfuricum	1.0
(18) *Talcum Crystallinum*	6.0

^a^ Each value (g) was represented as dry weight. Human take 9 g daily.

GLUT4 has been shown to play a key insulin-regulated glucose transporter expressed mainly in skeletal muscle and adipose tissue [12,13]. Impairment of GLUT4 expression, GLUT4 translocation and/or insulin signaling may influence insulin-stimulated glucose uptake, which lead to insulin resistance and hyperglycemia [14,15]. Therefore, increases of GLUT4 contents and/or translocation to the plasma membrane had long been regarded as a potential target in the management of diabetes mellitus.

AMP-activated protein kinase (AMPK) has also been shown to regulate GLUT4 translocation [16]. AMPK is a major cellular regulator of lipid and glucose metabolism [17]. AMPK is demonstrated to regulate a variety of different metabolic pathways, and thus has been recognized as a useful target for the management of metabolic disorders including type 2 diabetes and dyslipidemia [18,19]. Because lipid and glucose metabolism is dysregulated in type 2 diabetes, AMPK modulators have been suggested to be promising therapies [20].

The C57BL/6J mouse is susceptible to high-fat (HF) diet-induced hyperlipidemia, obesity and Type 2 diabetes [21]. However, the antidiabetic and antihyperlipidemia activity of BO is not well defined in high-fat-fed mice. Phosphorylation of Thr 172 of α subunits is essential for AMPK activity [22]. In this study, we investigated whether BO activated AMPK in liver tissue and skeletal muscle. As one of the possible mechanisms of action, we also evaluated the effect of BO on expressions of targeted genes involved in antidiabetes, antihyperlipidemia and lipogenesis in liver tissue, including phosphoenol pyruvate caboxykinase (PEPCK), glucose 6-phosphoatase (G-6Pase), peroxisome proliferator-activated receptor α (PPARα), sterol regulatory element binding protein 1c (SREBP1c) and fatty acid synthase (FAS).

## 2. Results

### 2.1. Oral Glucose Tolerance Test

**Figure 1:**
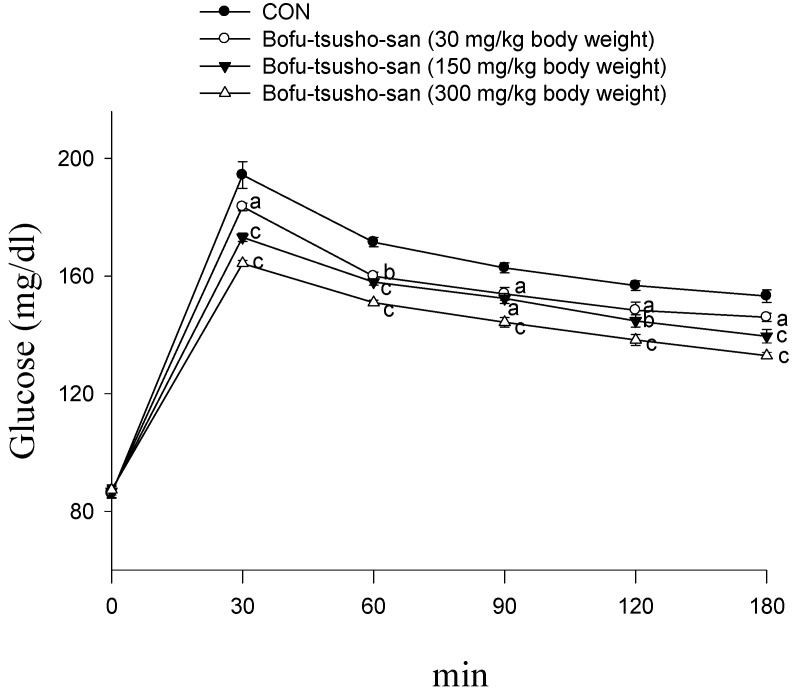
Effects of extract granules of Bofu-tsusho-san (BO) on oral glucose tolerance in ICR normal mice. Animals in all groups received oral glucose 30 min after the extract administration. Blood samples were collected and centrifuged at 3000 rpm for 10 min. Each point is the mean ± S.E. of 5 separate mice. ^a^ *p* < 0.05, ^b^ *p* < 0.01, ^c^ *p* < 0.001 significantly different compared with the control group in the same time by ANOVA.

Following treatment with BO (including 30, 150 and 300 mg/kg body weight), the levels of blood glucose were significantly decreased at 30, 60, 90 and 120 min glucose-loading as compared with the control (Figure 1).

### 2.2. Body Weight, Body Weight Gain and Food Intake

**Figure 2:**
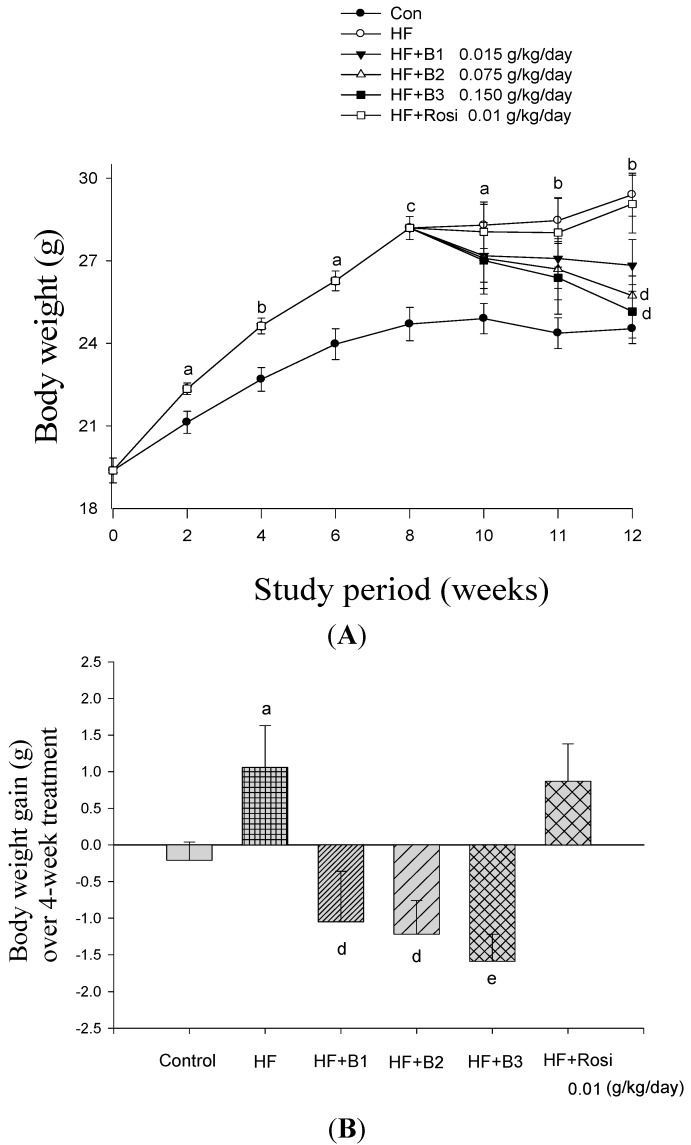

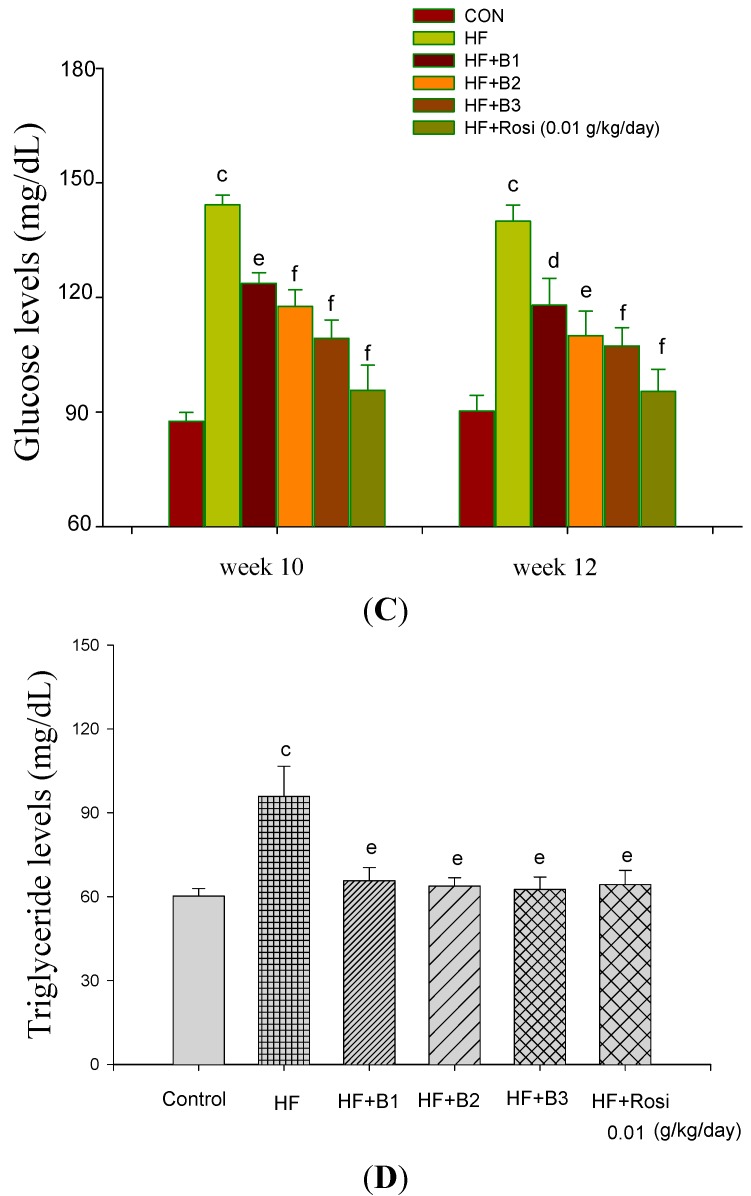
Effects of Bofu-tsusho-san (BO) on (**A**) body weight; (**B**) body weight gain; (**C**) glucose levels at week 10 and 12 and (**D**) plasma triglyceride level at week 12. Mice were fed with 45% high-fat diet (HF) or low-fat diet (CON) for 12 weeks. After 8 weeks, the HF mice were treated with vehicle, or B1, B2, B3, extract granules of BO (0.015, 0.075 and 0.150 g/kg body weight), or rosiglitazone (Rosi) (0.01 g/kg body weight) or vehicle accompanied with HF diet for 4 weeks. Visceral fat: EWAT + RWAT; EWAT, epididymal white adipose tissuel; RWAT: retroperioneal white adipose tissue. All values are means ± S.E. (*n* = 9). ^a^ *p* < 0.05, ^b^ *p* < 0.01, ^c^ *p* < 0.001 compared with the control (CON) group; ^d^ *p* < 0.05, ^e^ *p* < 0.01, ^f^ *p* < 0.001 compared with the high-fat + vehicle (distilled water) (HF) group by ANOVA.

All group mice started with similar mean body weights (19.38 ± 0.45 g). At the end of study, the body weight and weight gain of the HF- treated group is significantly greater than the CON group (*p* < 0.01, *p* < 0.05, respectively) (Figure 2A,B). B2- and B3- treated mice exhibit reduced body weight as compared with the HF group (*p* < 0.05, *p* < 0.05, respectively). All the BO- treated mice displayed resistance to body weight gain. The 4-week average food intake (g/mouse/day) of the HF group is much less than the CON group (*p* < 0.05). There are no significant differences in food intake (kcal/mouse/day) between the HF group and CON group. All the BO- and Rosi- treated mice did not differ from HF mice in food intake (including g/mouse/day and kcal/mouse/day). BO- treated mice and HF littermates consume high-fat diets similarly (g and kcal) (Table 2).

### 2.3. Tissue Weight

High-fat diets cause increases in weights of absolute adipose tissue (epididymal WAT 136.5%, mesenteric 37.4%, retroperitoneal WAT 231.0% and visceral fat 158.0%) (Table 2). The decreased body weight gain primarily reflects reduced fat accumulation with decreased weight of epididymal WAT as well as lowered visceral fat and retroperitoneal WAT in B1-, B2- and B3- treated mice.

### 2.4. Blood Glucose Levels

At week 10 and 12, HF-diet induced elevations in blood glucose levels (*p* < 0.001, *p* < 0.001, respectively). All the BO- and Rosi-treated groups displayed decreased blood glucose levels compared with the HF group (Figure 2C).

**Table 2:** Effects of Bofu-tsusho-san (BO) on absolute tissue weight, food intake, liver lipids and blood profiles. All values are means ±S.E. (*n* = 9). ^a^ *p* < 0.05, ^b^ *p* < 0.01, ^c^ *p* < 0.001 compared with the control (CON) group; ^d^ *p* < 0.05, ^e^ *p* < 0.01, ^f^ *p* < 0.001 compared with the high-fat + vehicle (distilled water) (HF) group. B1, B2, B3, extract granules of Bofu-tsusho-san (0.015, 0.075 and 0.150 g/kg body weight). BAT, brown adipose tissue; RWAT, retroperioneal white adipose tissue; MWAT, mesenteric white adipose tissue; Visceral fat was defined as the sum of epididymal and retroperioneal WAT; FFA, plasm free fatty acid. ^g^ Dose (g/kg/day).

Parameter	CON	HF	HF + B1	HF + B2	HF + B3	HF + Rosi
			0.015 ^g^	0.075 ^g^	0.150 ^g^	0.01 ^g^
**Absolute tissue weight (g)**						
EWAT	0.427 ± 0.025	1.010 ± 0.119 ^c^	0.671 ± 0.053 ^d^	0.544 ± 0.108 ^f^	0.569 ± 0.078 ^e^	0.604 ± 0.084 ^d^
MWAT	0.380 ± 0.018	0.522 ± 0.045 ^a^	0.424 ± 0.013	0.373 ± 0.039 ^d^	0.345 ± 0.050 ^e^	0.339 ± 0.038 ^e^
RWAT	0.126 ± 0.014	0.417 ± 0.041 ^c^	0.228 ± 0.035 ^e^	0.246 ± 0.044 ^d^	0.186 ± 0.032 ^f^	0.237 ± 0.051 ^d^
Visceral fat	0.553 ± 0.059	1.427 ± 0.161 ^c^	0.899 ± 0.074 ^d^	0.890 ± 0.164 ^d^	0.755 ± 0.115 ^e^	0.841 ± 0.146 ^d^
BAT	0.042 ± 0.003	0.041 ± 0.003	0.038 ± 0.006	0.044 ± 0.004	0.043 ± 0.003	0.052 ± 0.003
Liver (g)	1.052 ± 0.030	0.999 ± 0.055	0.975 ± 0.068	0.914 ± 0.052	0.988 ± 0.037	0.930 ± 0.022
Spleen	0.117 ± 0.007	0.119 ± 0.017	0.136 ± 0.011	0.150 ± 0.018	0.130 ± 0.016	0.123 ± 0.005
weight gain (g)	−0.21 ± 0.25	1.06 ± 0.57 ^a^	−1.05 ± 0.69 ^d^	−1.22 ± 0.46 ^d^	−1.59 ± 0.37 ^e^	0.87 ± 0.51
Food intake (g/mouse/day)	2.77 ± 0.06	2.37 ± 0.04 ^c^	2.21 ± 0.06	2.18 ± 0.06	2.23 ± 0.08	2.26 ± 0.04
Food intake (kcal/mouse/day)	9.36 ± 0.19	9.82 ± 0.21	9.19 ± 0.28	9.14 ± 0.29	9.27 ± 0.27	9.37 ± 0.19
Liver lipids						
total lipid (mg/g)	57.6 ± 2.8	97.2 ± 6.0 ^c^	71.6 ± 3.8 ^e^	68.0 ± 4.5 ^e^	65.4 ± 4.2 ^e^	63.8 ± 5.9 ^e^
Triacylglycerol (μmol/g)	35.6 ± 3.7	80.3 ± 7.3 ^c^	57.3 ± 5.5 ^e^	44.5 ± 4.7 ^f^	43.7± 6.6 ^f^	48.4 ± 5.4 ^f^
Blood profiles						
FFA (meq/L)	0.42 ± 0.03	0.93 ± 0.06 ^c^	0.57 ± 0.07 ^e^	0.51 ± 0.03 ^e^	0.39 ± 0.04 ^f^	0.59 ± 0.04 ^d^
TC (mg/dL)	90.5 ± 4.0	143.0 ± 3.1 ^c^	127.0 ± 4.1	124.7 ± 3.9 ^d^	121.2 ± 4.4 ^e^	115.0 ± 6.3 ^f^
Leptin (μg/mL)	1.24 ± 0.35	5.22 ± 0.94 ^b^	3.24 ± 0.53 ^e^	2.65 ± 0.49 ^e^	2.50 ± 0.29 ^f^	2.90 ± 0.56 ^e^
Adiponectin (μg/mL)	9.98 ± 0.36	7.13 ± 0.65 ^b^	9.69 ± 0.66 ^e^	10.44 ± 0.61 ^f^	10.86 ± 0.45 ^f^	12.25 ± 0.71 ^f^
Insulin (μg/L)	0.61 ± 0.04	0.93 ± 0.12 ^a^	0.76 ± 0.28	0.61 ± 0.19 ^d^	0.58 ± 0.06 ^d^	0.53 ± 0.04 ^d^

### 2.5. Blood Parameter, Leptin, Adiponectin and Insulin Levels in Blood and Liver Lipid

High-fat diets caused increased in blood TG, FFA and leptin (*p* < 0.001, *p* < 0.001, *p* < 0.001, respectively) (Figure 2D and Table 2). These substances are significantly lowered in all the BO- and Rosi- treated mice. High-fat diets exhibited enhanced in blood insulin and TC (*p* < 0.05, *p* < 0.001, respectively) (Table 2). B2-, B3- and Rosi- treated groups suppressed the increases in blood levels of TC and insulin. HF-feeding induced decreased blood levels of adiponectin. All the BO- and Rosi- treated groups displayed increased adiponectin levels. The liver total lipids and triacylglycerol concentrations were 68.8% and 125.6% greater, respectively, in the HF group than in the CON group. The hepatic total lipids and triacylglycerol concentrations displayed significantly lowered in B1-, B2-, B3- and Rosi- treated groups.

### 2.6. Histopathology of Adipose and Liver Tissue

**Figure 3:**
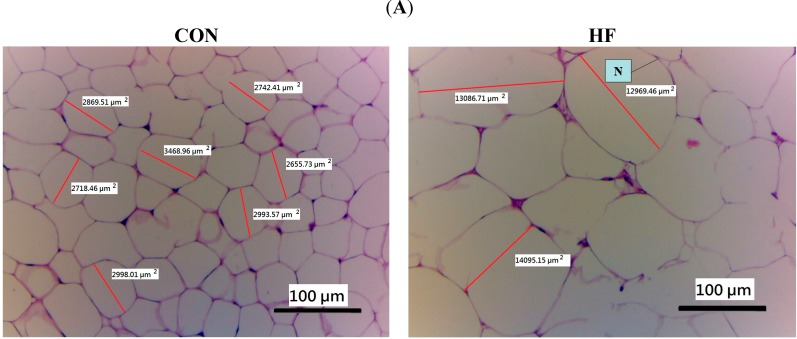

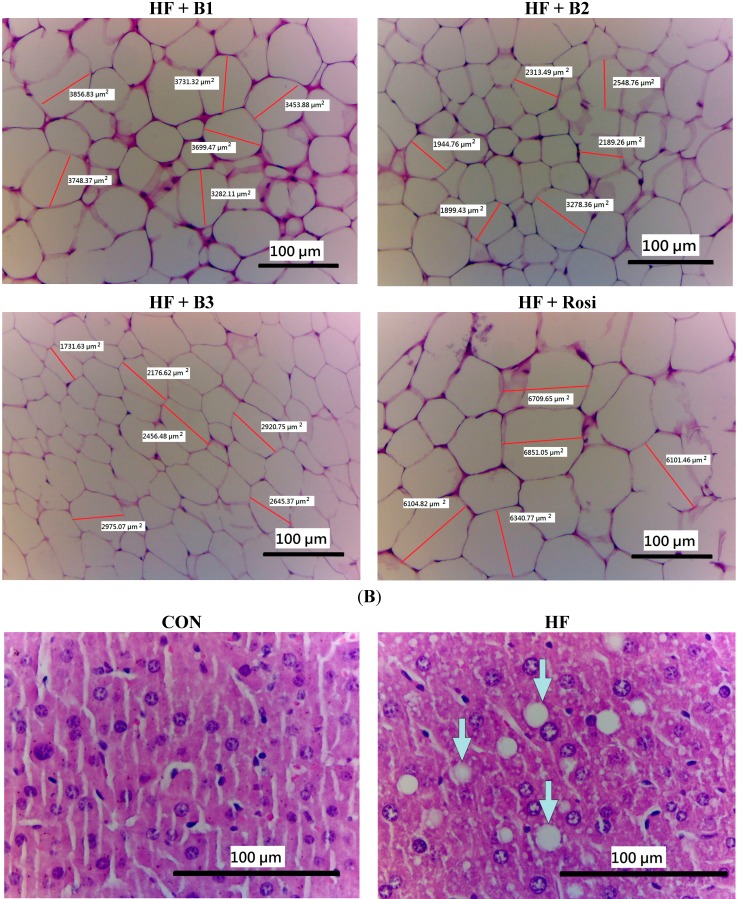

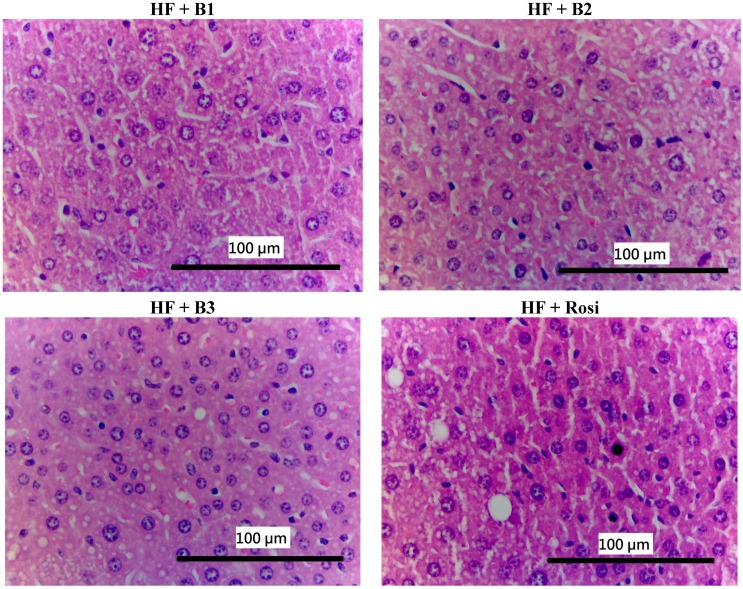
Effects of Bofu-tsusho-san (BO) on (**A**) epididymal WAT and (**B**) liver tissue morphology in the low-fat (CON), high-fat (HF), HF + B1, HF + B2, HF + B3, or HF + Rosi groups. Pictures of hematoxylin and eosin-stained sections of (**A**) epididymal adipocytes (Magnification: 10 (ocular) × 10 (object lens) from mice fed with BO. White adipose tissue (named adipocytes) is polyhedral by H&E stain. Due to embedded in paraffin as immersed in lipid solvents and could remove all the fats, the appearance of adipocyte showed string-like cytosol surrounding a vacuole. It was carefully observed unobvious nucleus (N) in the other side of cells; and (**B**) liver tissue (Magnification: 10 (ocular) × 20 (object lens)) from mice fed with BO. The high-fat diet induced the hepatic ballooning degeneration in the HF group as compared with the CON group. The ballooning degeneration is a form of liver parenchymal cell death and the nucleolus was squeezed into the other side named balloon (as the arrow indicated). This may be due to the heap of glycogen in the nucleus. High-fat diet induced obesity and insulin resistance. Insulin levels affected the storage of hepatic glycogen. Treatment with B1, B2 and B3 significantly decreased the degree of ballooning degeneration. Each presented is typical and representative of nine mice. Each presented is typical and representative of nine mice. B1, B2, B3, extract granules of BO (0.015, 0.075 and 0.150 g/kg body weight); Rosi: rosiglitazone (0.01 g/kg body weight).

Feeding the HF diet induced hypertrophy of the adipocytes compared with the CON group in epididymal WAT. The average area of the cut of the adipocytes in the HF group (13,383.77 µm^2^) is larger than in the CON group (2920.95 µm^2^). Treatment with B1 (3628.66 µm^2^), B1 (2362.26 µm^2^) and B3 (2154.65 µm^2^) significantly decreased the hypertrophy (Figure 3A). The average area of the cut of the adipocytes in the Rosi-treated group is (6421.55 µm^2^). The ballooning phenomenon in liver is visible on HF-diet. Following treatment with B1, B2, B3 and Rosi, these mice displayed significantly decreased the ballooning phenomenon than do HF mice (Figure 3B).

### 2.7. Target Gene Expressions in Liver Tissue

**Figure 4:**
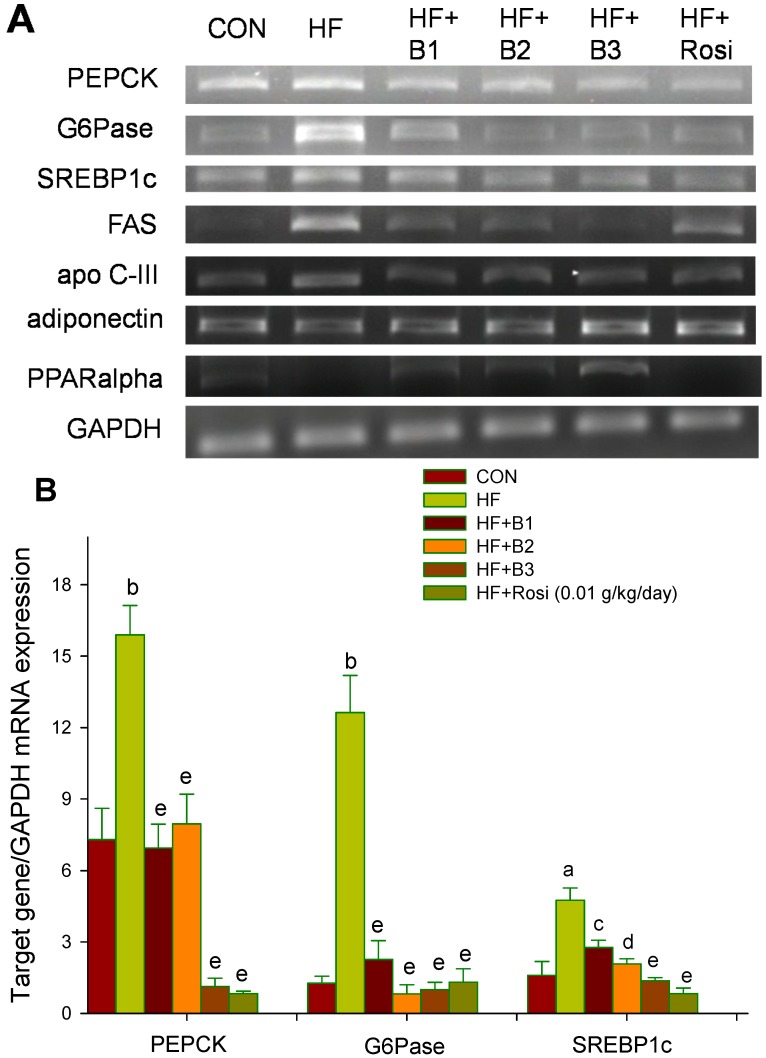

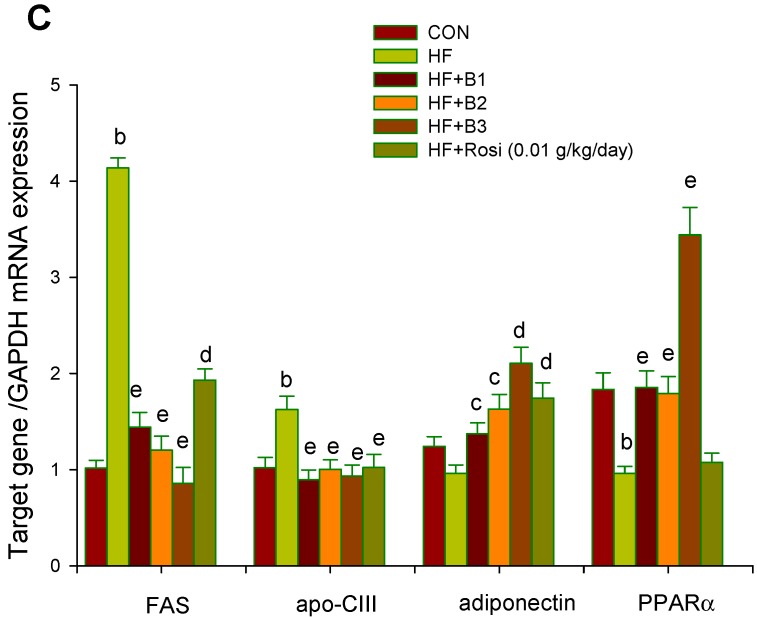
Semiquantative RT-PCR analyses including (**A**) image; (**B**) and (**C**) statistical data for phosphenolpyruvate carboxykinase (PEPCK), glucose-6-phosphatase (G6Pase), sterol regulatory element binding protein 1c (SREBP1c), fatty acid synthase (FAS), apo C-III, adiponectin and peroxisome proliferator-activated receptor α (PPARα) mRNA expression in liver tissue of the mice by oral gavage extracts of Bofu-tsusho-san (BO) or Rosi: rosiglitazone (0.01 g/kg body weight) or vehicle for 4 weeks. Total RNA (1 μg) isolated from tissue was reverse transcripted by MMLV-RT, 10 μL of RT products were used as templates for PCR. The expression levels of PEPCK, G6Pase, DGAT2, SREBP1c, PPARα, FAS, apo C-III and adiponectin mRNA were measured and quatified by image analysis. Values were normalized to GAPDH mRNA expression. All values are means ± S.E. (*n* = 9). ^a^ *p* < 0.01, ^b^ *p* < 0.001 compared with the control (CON) group; ^c^ *p* < 0.05, ^d^ *p* < 0.01, ^e^ *p* < 0.001 compared with the high-fat + vehicle (distilled water) (HF) group by ANOVA. B1, B2, B3, extract granules of BO (0.015, 0.075 and 0.150 g/kg body weight).

The mRNA levels of PEPCK, G6Pase, SREBP1c, FAS and apo C-III were higher in the HF group than in the CON group (*p* < 0.001, *p* < 0.001, *p* < 0.001, *p* < 0.01, *p* < 0.001, *p* < 0.001, *p* < 0.001, respectively), whereas the mRNA levels of PPARα were decreased in the HF group (*p* < 0.001). Treatment with B1-, B2-, B3- and Rosi- treated groups significantly decreased the mRNA level of PEPCK, G6Pase, SREBP1c, FAS and apo C-III, while increased the hepatic adiponectin mRNA level compared with the HF group (*p* < 0.05, *p* < 0.05, *p* < 0.01, *p* < 0.01, respectively). All the BO- treated groups increased the mRNA level of PPARα (Figure 4).

### 2.8. The Protein Contents of GLUT4 in Skeletal Muscle and Phospho-AMPK (Thr172) in Skeletal Muscle and Liver Tissue

**Figure 5:**
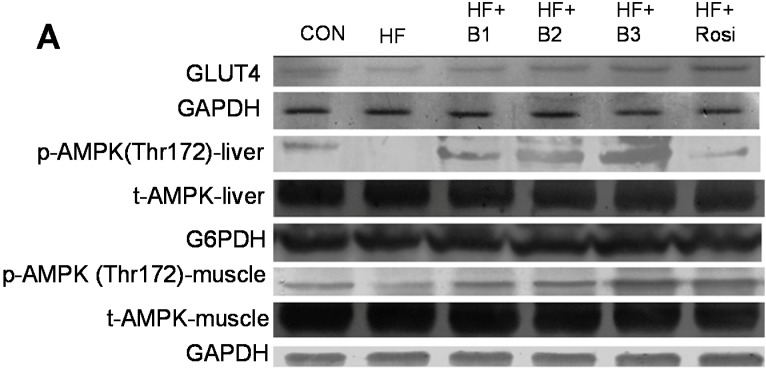

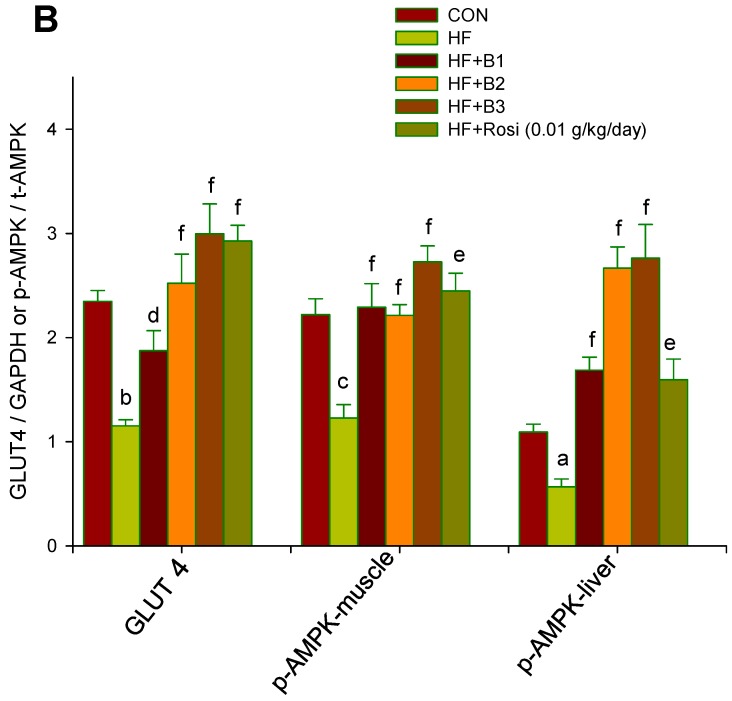
The protein contents of glucose transporter 4 (GLUT4) in skeletal muscle, the ratio of phospho-AMPK (Thr172) to total AMPK in liver tissue and skeletal muscle of the mice by oral gavage Bofu-tsusho-san (BO), rosiglitazone (Rosi) (0.01 g/kg body weight) or vehicle for 4 weeks. Protein was separated by 12% SDS-PAGE detected by Western blot including (**A**) image and (**B**) analysis data. All values are means ± S.E. (*n* = 9). ^a^ *p* < 0.05, ^b^ *p* < 0.01, ^c^ *p* < 0.001 compared with the control (CON) group; ^d^ *p* < 0.05, ^e^ *p* < 0.01, ^f^ *p* < 0.001 compared with the high-fat + vehicle (HF) group by ANOVA. B1, B2, B3, extract of BO.

High-fat diets elicit lower levels of skeletal muscular GLUT4 proteins in HF group than in CON group (*p* < 0.01). The B1-, B2-, B3 and Rosi- treated mice exhibited increased GLUT4 proteins in skeletal muscle (*p* < 0.05, *p* < 0.001, *p* < 0.001, *p* < 0.001, respectively). High-fat diets caused much less levels of phosphorylated AMPK protein in HF mice than in CON mice in skeletal muscle and liver tissue (*p* < 0.001, *p* < 0.05, respectively). The B1-, B2-, B3- and Rosi- treated mice displayed increased levels of phosphorylated AMPK both in skeletal muscle and liver tissue (Figure 5).

## 3. Discussion

This study demonstrated that BO treatment displayed reduced blood glucose levels but also effectively improved lipid metabolism. Decreased blood glucose levels should be associated with improved glucose uptake. We monitored the uptake of glucose into muscle and found that BO increased the contents of GLUT4 and caused glucose uptake into the peripheral tissues, thus had favorable effects on glucose levels. Further, BO increased the phosphorylation of AMPK in both liver and skeletal muscle. These findings are involved in the results on GLUT4 contents and AMPK activation.

High-fat (HF) diets induce obesity, hyperglycemia, insulin resistance, and fatty liver in C57BL/6J mice [23]. HF diet is well-known to increase body weight, body fat and induce insulin resistance and elevated levels of triglycerides [21]. Therefore, a HF-fed mouse model was chosen to address hypolipidemic and hopoglycaemic properties of BO.

In the present study, feeding C57BL/6J mice with HF diet induced hyperglycemia, obesity, hyperinsulinemia, hypertriglycemia, hyperleptinemia and excess circulating free fatty acid, consistent with an earlier report [21]. Following treatment with BO, blood glucose, circulating triglycerides, and visceral fat mass were lowered as well as a reduction in free fatty acid (FFA), thus improved insulin resistance and lipid profiles. Since visceral obesity is thought to play a major role in the pathogenesis of metabolic syndrome [24], BO is likely to be useful in the treatment of metabolic syndrome associated with visceral adiposity, such as hyperlipidemia, insulin resistance, and Type 2 diabetes.

AMPK is known to regulate a variety of different metabolic pathways, been recognized as a useful target for the treatment of metabolic disorders such as T2D and dyslipidemia [20]. Therefore, our findings regarding activation of AMPK in both liver and skeletal muscle implicate BO as a phytonutrients with therapeutically potential for diabetic and dyslipidaemic disorders by targeting AMPK.

To explore antidiabetic properties of BO, we measured GLUT4 proteins in skeletal muscle as the major tissues, which is the major tissue responsible for insulin-mediated glucose utilization. In the present study, high-fat diets cause decreases in GLUT4 proteins, which is reversed by treatment with B1, B2, B3 and Rosi. The increased protein contents of GLUT4 indicated that BO and Rosi improved glucose utilization in skeletal muscle by restoring translocation GLUT4 to plasma membrane.

Further, the AMPK activator AICAR has been shown to lower plasma glucose and ameliorate insulin resistance in animal studies [25,26]. AMPK activation is regared as another major regulator of GLUT4 translocation during exercise or in response to some antidiabetic agents such as AICAR, metformin [17]. To ascertain whether AMPK activation is involved, we monitored phosphorylation of AMPK in skeletal muscle of BO- and Rosi- treated mice and observed a pronounced enhancement. Whether increases in phosphorylated AMPK levels interfere with stimulation of GLUT4 contents are needed to shed light on phosphorylation of AS160 pathway.

Hepatic glucose overproduction is a crucial factor in diabetic hyperglycemia. PEPCK is a key rate-limiting enzyme of gluconeogenesis. G-6Pase plays a role in glucose homeostasis [27]. The activities of G-6Pase increased significantly in the liver of diabetic rats [28]. The expressions of these enzymes including PEPCK and G-6Pase were decreased by BO, suggesting that down-regulation of PEPCK and G-6Pase contributes to the antidiabetic effect of BO.

Glycyrrhizin has been shown to could ameliorate insulin resistance, dyslipidemia in fructose-induced metabolic syndrome in rat model including decreased the enhanced levels of blood glucose, insulin and lipids and displayed elevated skeletal muscular GLUT4 proteins [29]. In this study, BO exerted decreased blood levels of glucose and triglycerides while increased GLUT4 proteins may be partly due to the effects of glycyrrhizin. The active principle of Glycyrrhizae Radix, glycyrrhizic acid, is demonstrated to decrease in blood levels accompanied by a decrease in PEPCK activities [30]. Geniposide has been shown to be an effective hypoglycemic agent in diabetic mice induced by a high-fat diet and streptozotocin, and the hypoglycemic effects of this compound may be mediated by inhibiting the glycogen phosphorylase and G6Pase mRNA expression and protein [31]. Our HPLC data in favor of the concept that geniposide and glycyrrhizin constituents contained in BO may be partly involved in glucose homeostasis. Moreover, another study showed that geniposide displayed hepatoprotective effects and decreased blood levels of triglycerides and free fatty acids and increased hepatic PPARα expressions in rats fed with a high-fat diet [32]. In this study, BO exerted decreased blood levels of glucose and triglycerides and hepatic G6Pase while increased PPARα expressions may be partly due to the effects of geniposide.

In this study, BO-induced increases in AMPK phosphorylation, indicating that BO lowered blood glucose by hepatic activation of AMPK, and inhibiting hepatic glucose production via PEPCK and G-6Pase down-regulation. Metformin is an established treatment for type 2 diabetes due to its ability to increase peripheral glucose uptake while reducing hepatic glucose production in an AMPK-dependent manner [33]. Collectively, this study demonstrated that BO act like metformin and caused glucose lowering by AMPK activation both in liver and muscle, in addition to its ability to increase glucose uptake in skeletal muscle, also by down-regulations of PEPCK and G-6Pase to inhibiting hepatic glucose production.

Circulating levels of adiponectin were increased in all BO- and Rosi- treated groups. TZDs exhibit as an AMPK activator but different from metformin, and reduced liver fat accumulation and ameliorated insulin resistance due to its effect on plasma adipocytokine (including adiponectin and leptin) and more AMPK activation [34]. Treatment diabetic mice with rosiglitazone exhibited the importance of adipose tissue-derived adiponectin in vascular and metabolic benefits [35]. Rosiglitazone elicits its protective functions against non-alcoholic fatty liver disease largely through the induction of adiponectin, which prevents mitochondria stresses by promoting GSK3beta activation and UCP2 upregulation, two pathways coordinating the glucose and lipid metabolism in liver [34]. Increased adiponectin may activate AMPK in various metabolic organs and tissues to lower glucose and lipids in diabetic DIO mice [36,37]. Previous study showed that adiponectin stimulates glucose utilization and fatty-acid oxidation by activating AMP- activated protein kinase [37]. Increased fatty acid oxidation results in decreased TG synthesis, which, in turn, lowered its accumulation in organs. Thus, adiponectin effects of BO may play a role in decreased hepatic TG. In addition, adiponectin improves glucose metabolism by regulating glucose uptake in skeletal muscle and gluconeogenesis in liver tissue [37,38]. Thus, there is one possibility that increased adiponectin plays a key role in regulating the effects of BO to improve glucose and lipid metabolism in DIO mice.

In the present study, BO- treated mice exhibit decreased body weight gain, which primarily reflects reduced fat accumulation with decreased weight of epididymal WAT as well as diminished weights of visceral fat. Blood leptin levels are markedly lowered in BO- treated mice, consistent with reduced fat mass, indicating that increased leptin sensitivity. Moreover, hepatic PPARα regulation suggested to be crucial in energy homeostasis, such as in body weight control and lipid metabolism [39]. A possible explanation for decreases in body weight gain is associated with laxatic effect of baicalein and senosides, sympathomimetic and thermogenesis activity of ephedrine [6], and its PPARα properties.

Blood triglycerides and free fatty acid are lowered in BO- and Rosi- treated groups. SREBP1c have been shown to up-regulate a number of lipogenic genes [40] and in PPARα- deficient mice, dysregulation of SREBP-mediated lipogenic genes have been shown [41], suggesting the role of PPARα in SREBP- mediated regulation of lipogenic genes. PPARα is highly expressed in liver and controls β- oxidation and hepatic PPARα activation may be crucial in lipid metabolism [42]. The present studies confirm that BO exerted lipid- lowering effects via regulation of genes expressions involved in lipid synthesis including SREBP1c and FAS. Moreover, BO treatment increased hepatic expressions of PPARα, whereas decreased the apo C-III expressions, which is accompanied with the decreases in plasma lipid level and hepatic lipid accumulation, indicating that BO regulates lipid metabolism in part due to PPARα regulation in liver tissue.

The morphological analysis showed that treatment with BO decreased the average area of the cut of adipocytes. Lipids that accumulate in adipose tissue are largely derived from circulating TG. The liver is a major organ to metabolize fat and for lipid and lipoprotein metabolism. Circulating TG is fluctuating; therefore, it is possible that BO may be able to mobilize fat from adipose tissue by increasing hepatic lipid catabolism including decreased TG synthesis while increased fatty acid oxidation in liver effectively regulated morphometric adipocytes.

## 4. Materials and Methods

### 4.1. HPLC Analysis

Bofu-tsusho-san extract granules were purchased from Sheng Foong Pharmaceutical Co., Ltd. (Taipei, Taiwan) in February 2013 and were authenticated by the Institute of Chinese Pharmaceutical Sciences, China Medical University. The high-performance liquid chromatographic (HPLC) was performed on a system equipped with HITACHI 7000 system (Shinko, Kobe, Japan) including Autosampler L-7200, L-7400 UV detector, Column oven L-7300 and Interface D-7000. Glycyrrhizin and geniposide were two reference makers applied for HPLC analysis. Chromatographic separation of these two makers was performed on a 5C_18_-AR-II column (4.6ID × 250 mm) maintained at ambient temperature (24 ± 1 °C). The mobile phase of HPLC analysis for glycyrrhizin consisted of a mixture of 2% acetic acid solution and acetonitrile (35:65). The mobile phase of HPLC analysis for geniposide consisted of a mixture of acetonitrile and H_2_O (13:87). The flow rate for HPLC analysis was 1.0 mL/min. The fraction was chromatographed to isolated glycyrrhizin and geniposide were 0.401%, 0.547% (Figures S1 and S2), respectively. The agent was diluted or adjusted to approximately 0.015, 0.075 and 0.150 g/kg body weight that was administrated orally to mice at volume of 1 mL/100 g body weight.

### 4.2. Animals and Experimental Design

All animal procedures were performed as per guidelines provided by the Institutional Animal Care and Use Committee of Central Taiwan University of Science and Technology. The study contained two parts of including part 1: Oral glucose tolerance test (OGTT). The ICR mice normal mice (*n* = 5) were fasted for 12 h but were allowed access to BO extract granules (including 0.030, 0.150 and 0.300 g/kg body weight) or an equivalent amount of normal vehicle (water) was given orally 30 min before an oral glucose load (1 g/kg body weight). Blood samples were collected from the retro-orbital sinus of fasting mice at the time of the glucose administration 0 min and every 30 min until 3 h after glucose administration to determine the levels of glucose. The part 2 animal study: the study was performed as shown as described [43]. C57BL/6J mice (4–5 weeks old) were purchased from the National Laboratory Animal Breeding and Research Center, National Science Council. Animals were maintained on a 12 h light/dark cycle (light cycle: AM7:00 to PM7:00). One week after acclimation, the C57BL/6J mice were divided randomly into two groups. The control (CON) group (*n* = 9) was fed low-fat diet (Diet 12450B, Research Diets, Inc., New Brunswick, NJ, USA), whereas the experimental group was fed a 45% high-fat diet (Diet 12451, Research Diets, Inc., New Brunswick, NJ, USA) for 12 weeks. The low-fat diet was composed of protein 20%, carbohydrate 70% and fat 10%, whereas high-fat diet was composed of protein 20%, carbohydrate 35% and fat 45% (of total energy, %kcal). After 8 week diet-induction period, the high-fat treated mice were randomly subdivided into 5 groups (*n* = 9 per group). BO extract granules (including 0.015, 0.075 and 0.150 g/kg body weight) or rosiglitazone (Rosi; 1% methylcellulose 10 mg/kg body weight, obtained from GlaxoSmithKline Product No: BRL49653 C) or vehicle were administrated through oral gavage one time per day from the 9th to 12th week, and the mice were still on the high-fat diet, while the CON and high-fat control (HF) mice were treated with vehicle only. The compositions of the experimental diets are shown as described [43]. At the end of the study, food is deprived from animal (from 10 p.m. to 10 a.m.). The next day, the mice were sacrificed for blood and tissue collection and analysis. Livers, skeletal muscles and white adipose tissues (WATs) (including epididymal, mesenteric and retroperitoneal WAT) were weighed and excised according to the defined anatomical landmarks, and followed by immediately frozen using liquid nitrogen, and then kept at −80 °C for the analysis of target gene expression. Heparin (30 units/mL) (Sigma, St. Louis, MO, USA) were added into blood sample. Plasma samples were collected by centrifugation at 1600× *g* for 15 min at 4 °C. The separation of the plasma was finished within 30 min. Plasma were obtained for insulin and leptin assay.

### 4.3. Measurement of Body Weight, Body Weight Gain and Food Intake

Body weight and food intake were monitored. Body weight was measured daily at the same time throughout the study. Body weight gain is defined as the differences between the body weight of the day and the next day. The pellet food was weighed and followed by placing in the cage food container. After 24 h, the remaining food was weighed, and the difference represented the daily food intake.

### 4.4. Blood Parameters Assay

Blood samples (0.8 mL) were collected from the retro-orbital sinus of fasting mice and the level of glucose was measured by the glucose oxidase method (Model 1500; Sidekick Glucose Analyzer; YSI Incorporated, Yellow Springs, OH, USA). Plasma triglycerides (TG), total cholesterol (TC) and free fatty acids (FFA) were analyzed using commercial assay kits according to the manufacturer’s directions (Triglycerides-E test, Cholesterol-E test and FFA-C test, Wako Pure Chemical, Osaka, Japan).

### 4.5. Adipocytokine and Insulin Levels Assay

The levels of insulin and leptin were analyzed by ELISA using a commercial assay kit according to manufacturer’s directions (Mouse/Rat Adiponectin ELISA kit, B-Bridge International, GmbH, Germany; mouse insulin ELISA kit, Sibayagi, Gunma, Japan and mouse leptin ELISA kit, Morinaga, Yokohama, Japan).

### 4.6. Histopathology of Adipose and Liver Tissue

Small pieces of epididymal WAT and liver tissue were fixed with formalin (200 g/kg) neutral buffered solution and embedded in paraffin. Sections (8 µm) were cut and stained with hematoxylin and eosin. For microscopic examination, a microscope (Leica, DM2500, Gallen, Switzerland) was used, and the images were taken using a Leica Digital camera (DFC-425-C, Gallen, Switzerland).

### 4.7. Measurement of Hepatic Lipids

Hepatic lipids were extracted using a previously described protocol [44]. For the hepatic lipid extraction, the 0.375 g liver samples were homogenized with 1 mL distill water for 5 min. Finally, the dried pellet was resuspended in 0.5 mL ethanol and analyzed using a triglycerides kit as used for serum lipids.

### 4.8. Isolation of RNA and Relative Quantization of mRNA Indicating Gene Expression

Total RNA from the liver tissue was isolated with a Trizol Reagent (Molecular Research Center, Inc., Cincinnati, OH, USA) according to the manufacturer’s directions. The integrity of the extracted total RNA was examined by 2% agarose gel electrophoresis, and the RNA concentration was determined by the ultraviolet (UV) light absorbency at 260 and 280 nm (Spectrophotometer U-2800A, Hitachi, Kanto, Japan). Total RNA (1 μg) was reverse transcribed to cDNA with 5 μL Moloney murine leukemia virus reverse transcriptase (Epicentre, Madison, WI, USA) as a previously described protocol [43]. The polymerase chain reaction (PCR) was performed in a final 25 μL containing 1 U Blend Taq™-Plus (TOYOBO, Japan), 1 μL of the RT first-strand cDNA product, 10 μΜ of each forward (F) and reverse (R) primer, 75 mM Tris-HCl (pH 8.3) containing 1 mg/L Tween 20, 2.5 mM dNTP and 2 mM MgCl_2_. The primers are shown in Table 3. The products were run on 2% agarose gels and stained with ethidium bromide. The relative density of the band was evaluated using AlphaDigiDoc 1201 software (Alpha Innotech Co., San Leandro, CA, USA). All the measured PCR products were normalized to the amount of cDNA of GAPDH in each sample.

**Table 3:** Primers used in this study.

Gene	Accession Number	Forward Primer and Reverse Primer	PCR Product (bp)	Annealing Temperature (°C)
Liver				
PEPCK	NM_011044.2	F: CTACAACTTCGGCAAATACC R: TCCAGATACCTGTCGATCTC	330	52
G6Pase	NM_008061.3	F: GAACAACTAAAGCCTCTGAAAC R: TTGCTCGATACATAAAACACTC	350	50
SREBP1c	NM_011480	F: GGCTGTTGTCTACCATAAGC R: AGGAAGAAACGTGTCAAGAA	219	50
FAS	NM_007988	F: TGGAAAGATAACTGGGTGAC R: TGCTGTCGTCTGTAGTCTTG	240	50
Adiponectin	NM_009605.4	F: TCTTCTACAACCAACAGAATCA R: GTATCATGGTAGAGAAGGAAGC	324	50.5
PPARα	NM_011144	F: ACCTCTGTTCATGTCAGACC R: ATAACCACAGACCAACCAAG	352	55
apoC-III	NM_023114.3	F: CAGTTTTATCCCTAGAAGCA R: TCTCACGACTCAATAGCTG	349	47
GAPDH	NM_031144	F: TGTGTCCGTCGTGGATCTGA R: CCTGCTTCACCACCTTCTTGA	99	55

### 4.9. Western Immunoblotting Analysis

Protein extractions and immunoblots for the determination of GLUT4 and phospho-AMPK (Thr172) proteins were carried out on frozen skeletal muscle and liver tissue from mice according to a previous report [45]. Briefly, liver tissue (0.1 g) was homogenized with lysis buffer (pH 6.4) and protease inhibitors. 40 μg of each homogenate is used for SDS-PAGE and immunoblotting as a previously described protocol [46]. Additionally, GLUT4 were carried out on frozen skeletal muscle from mice briefly described: Skeletal muscle (1 g) was powdered under liquid nitrogen and homogenized for 20 s in buffer (pH 7.4). The total membrane fractions were collected with buffer and centrifuged as a previously described protocol [47,48]. The protein contents of GLUT4 (Santa Cruz Biotechnology, Texas, CA, USA), phospho-AMPK (Abcam Inc, Cambridge, MA, USA) and total AMPK (Cell signaling Technology, Inc., Danver, MA, USA) were detected by immunoblotting using a rabbit polyclonal antibody. The protein concentration in supernatant was determined with a BCA protein assay kit (Thermo Scientific, Rockford, IL, USA). The membranes were blocked with 5% slim milk in Tris-buffered saline (TBS) (Amershan BioSciences, Uppsala, Sweden) containing 0.05% Tween-20 (Bio Rad, Hercules, CA, USA) and incubated overnight at 4 °C with anti-GLUT4 and anti-phospho-AMPK at 1:200 dilution and anti-total-AMPK at 1:1000 dilution. Subsequently, the membranes were washed three times with TBS containing 0.05% Tween-20 and incubated with secondary antibody anti-rabbit (1:1000) (Jackson Immuno Research, Laboratories, Inc., West Grove, PA, USA) for 1 h. Immunoreactive bands were detected with ECL reagent kit (GE Healthcare BioSciences, Buckinghamshire, UK). The density blotting was analyzed using Alpha Easy FC™ software (Alpha innotech corporation, Randburg, South Africa). Structural proteins GAPDH (Santa Cruz Biotecnology, Texas, CA, USA) and β-actin (Santa Cruz Biotecnology, Texas, CA, USA) were obtained by stripping the nitrocellulose membrane proteins of liver and skeletal muscle.

### 4.10. Statistical Analysis

Data were expressed as mean ± S.E. values. Whenever possible, data were subjected to analysis of variance, followed by Dunnett’s multiple range tests, using SPSS software (SPSS Inc., Chicago, IL, USA). *p* < 0.05 was considered to be statistically significant.

## 5. Conclusions

This study revealed for the first time that effects and molecular mechanisms of BO regarding decreased glycaemia and dyslipidaemia after a 4-week treatment in HF-fed mice. BO increased AMPK phosphorylation (activated AMPK) in both skeletal muscle and liver tissue. The hypoglycemic effect of BO is associated with increased GLUT4 protein contents to elevate glucose uptake in skeletal muscle; on the other hand, inhibition of hepatic glucose production (down-regulated PEPCK and G-6Pase expression). Further, BO activated hepatic AMPK, and increased fatty acid oxidation (PPARα), while decreased lipogenic enzyme expression (including down-regulated SREBP1c and FAS expression), which contributed to the lowering of circulating triglycerides. This biological function of BO might be associated with the increased activity of AMPK. These findings provide new insights into understanding that BO exerted antidiabetic and antihyperlipidaemic activity in HF-fed mice.

## Supplementary Materials

Supplementary materials can be found at http://www.mdpi.com/1422-0067/15/11/20022/s1.
